# Role of Complex Networks for Integrating Medical Images and Radiomic Features of Intracranial Ependymoma Patients in Response to Proton Radiotherapy

**DOI:** 10.3389/fmed.2019.00333

**Published:** 2020-01-17

**Authors:** Marco Dominietto, Alessia Pica, Sairos Safai, Antony J. Lomax, Damien C. Weber, Enrico Capobianco

**Affiliations:** ^1^Center for Proton Therapy, Paul Scherrer Institute, Villigen, Switzerland; ^2^Radiation Oncology Department, University Hospital of Bern, Bern, Switzerland; ^3^Center for Computational Science, University of Miami, Coral Gables, FL, United States

**Keywords:** ependymoma, medical imaging, radiomics, precision medicine, therapy response, network inference

## Abstract

Human cancers exhibit phenotypic diversity that medical imaging can precisely and non-invasively detect. Multiple factors underlying innovations and progresses in the medical imaging field exert diagnostic and therapeutic impacts. The emerging field of radiomics has shown unprecedented ability to use imaging information in guiding clinical decisions. To achieve clinical assessment that exploits radiomic knowledge sources, integration between diverse data types is required. A current gap is the accuracy with which radiomics aligns with clinical endpoints. We propose a novel methodological approach that synergizes data volumes (images), tissue-contextualized information breadth, and network-driven resolution depth. Following the Precision Medicine paradigm, disease monitoring and prognostic assessment are tackled at the individual level by examining medical images acquired from two patients affected by intracranial ependymoma (with and without relapse). The challenge of spatially characterizing intratumor heterogeneity is tackled by a network approach that presents two main advantages: (a) Increased detection in the image domain power from high spatial resolution, (b) Superior accuracy in generating hypotheses underlying clinical decisions.

## Introduction

### Background

Modern medical imaging methods are increasingly used in the clinical practice to visualize rich hierarchies of anatomic details in a variety of tissues and organs. By combining contrast agents and dynamic acquisitions, maps of images can show the complex interdependence of pathophysiological processes characterizing various diseases. This is especially important in oncology. Many factors influence tumor progression, namely hemodynamic, metabolism, pH, shape, etc. (1–4). These factors deliver information useful for planning treatment and follow-up processes. The quality of the results of such processes depends on an efficient use of standard clinical imaging scanners like MRI (magnetic resonance imaging), PET (positron emission tomography), and CT (computer tomography).

Past research efforts were directed mostly toward the search for individual biomarkers. Ideally, such biomarkers were conceived as targets of specific drugs. However, this strategy clearly implies an oversimplification of reality. Tumors are composed by non-homogeneous tissue, and the component tissues naturally communicate in a complex way between them, with the hosting organ and with the rest of the body (5). Additionally, having an unprecedented amount of information leads to a paradox: our ability to produce highly detailed data may still be insufficient for an accurate detection due to redundancies, spurious correlations, missing data, algorithmic approximations etc.

### Precision Medicine Focus

Precision Medicine (PM) is an approach conceived for disease treatment and prevention taking specifically into account individual variability in a variety of health-influencing factors (6). Naturally enough, the aim from a data science perspective is to integrate all multisource information for personalized management. Data-driven PM advances leverage data aggregation, pattern detection, feature extraction and similar strategies aimed at improving outcome management and quality of care. Among the goals of data science there are: (1) Individual profiling, diagnostic choices, treatment decisions, prognostication and (2) Integration of disparate information identifying clinically relevant associations, early signals of disease onset or progression and changes in health trajectories.

Through radiomics, radiographic images are fuel for PM [see (7), among others]. The value of image-associated clinical details is increasingly contributed by automated learning via computer aided algorithms. An example is deep learning (DL). DL is expected to leverage large-scale collections of images to extract clinical aspects not immediately and/or clearly associated to a clinical question. Despite being efficient, Dl is known to be not exempt from hurdles of various nature (8, 9). Therefore, as medical images span informative details far beyond the possible answers to any specific clinical question, next generation computational inference tools (DL and other types) should be tuned to the assembling and processing of information while preserving interpretability of results.

### Rationale

In cancer research, data types are generated to cover global as well as particular characteristics (or features) of tumors. Nonetheless, we can hardly achieve comprehensive summaries or maps of tumors. It is also problematic to establish linkages between features, making the task of predicting tumor progression itself remains only partially accomplished. Clearly enough, the prediction of tumor outcome following treatment remains a major goal of clinicians as well as the key feedback for patients. This is again in line with the PM shift of paradigm (10). Two objectives are: (a) to provide data syntheses and (b) to establish linkages. The added value with instruments like networks is the identification of significant associations within well-characterized disease contexts. The way to look at the cancer puzzle is neither unique nor universal. We want to stress the role of specific features, as explained in the next section.

### Our Contribution

Data connectivity in radiomics was discussed in Dominietto et al. (11). In principle, connected biomarkers open new avenues to therapeutic paths, and allows assessment of emerging digital biomarkers (12). Here, we demonstrate the applicability of a PM network approach in onco-radiomics (13, 14). We considered patients affected by intracranial ependymoma whose response to radiotherapy was assessed by MRI. Identifying secondary spots in the presence of primary tumor is typically done in cancer radiology, and the recourse to MRI methods may be justified for instance by the need of identifying tumor regions with abnormal vascularity (15). Intra-tumor heterogeneity indicates the need of integrating physiological signals, typically metabolism or proliferation, and achieving more accurate description (16). It remains challenging to spatially characterize heterogeneous tissue with tumor marks and treatment-related changes. This information should be integrated over time to improve predictive models for patient outcome (17).

The value of the proposed analysis is toward our understanding of the potential of a network inference approach within the radiomics [see also (11, 12) for treatment of the topic]. From the image domain, specific features were selected from repeated measurements (MRI) and mapped onto a network domain. By preserving the spatial resolution present in voxel domain, the mapping offers various advantages: (a) Marks/patterns indicating possible disease relapse can be detected, which adds predictive value with regard to the assessment of therapy effects; (b) Timely therapeutic intervention strategies can be topologically analyzed and monitored; (c) With more patients recruited generalization of the approach may provide patient stratifications and disease classifications of relevant to clinical trials design.

## Materials and Methods

### Image Features in Ependymoma Studies

Two young patients affected by ependymoma have been considered for this study. The first patient is a 12-year old female presented with a 3-month history of headache. Brain MRI depicted a solid mass in the left occipital lobe. No metastatic lesions were found at image workup. The child was submitted to gross total resection of the tumor; pathological diagnosis was of a WHO grade III anaplastic ependymoma. The patient received local proton therapy (PRT) according the HIT-2000 protocol guidelines. The dose of PRT was of 59.4Gy (RBE), 1.8Gy (RBE) par fraction. PRT was delivered with pencil beam scanned protons technique. After definitive treatment, the patient was followed with routine physical exams and serial MRI. Three years later, a tumor recurrence was observed at the primary site. The patient was re-operated (total resection). New pathological exam confirmed WHO grade III anaplastic ependymoma recurrence. The patient received a second course of PRT with pencil beam scanned protons technique to the site of recurrent disease. The gross tumor volume included the tumor bed after the second surgery. The clinical target volume margin was ≤0.5 cm adjusted to anatomical boundaries and to the optical tract. A planning target volume of 4 mm was applied. The targets were treated to 59.4 Gy (RBE), delivered in 1.8 Gy (RBE) fractions on 5 days each week.

The second patient is a 14-year old male presented with neck pain, confusion with word finding disorder, blurred speech and ophthalmological visual field defect. MRI showed a solid and polycystic mass in the right occipital lobe with an important edema surrounding the lesion. The child underwent gross total resection of the tumor and pathological diagnosis was a WHO grade III anaplastic ependymoma. The patient received local PRT of 59.4 Gy (RBE), 1.8 Gy (RBE) par fraction with pencil beam scanned protons technique. Also, in this case, after the radiotherapy treatment, the patient was followed with routine physical exams and serial MRI. At this time, 2 years after the end of treatment, no recurrences have been observed. In both cases, before surgery, a consistent set of MRI acquisitions has been acquired in order to define tumor staging and plan the surgical and radiation therapy treatment. From this set of acquisitions, 21 anatomical and physiological features were extracted. They are reported in Table 1.

**Table 1:** List of anatomical and physiological features acquired using MRI (details in the footnote a-e).

**Type**	**Feature n**.	**MRI acquisitions**
Anatomical structure	1	HiRes 3D T1w gradient echo pre-CA
	2	HiRes 3D T1w gradient echo post-CA
	3	T1w fluid suppression pre-CA
	4	T1w fluid suppression post-CA
	5	T2w coronal
	6	T2w transaxial
	7	T2w sagittal
	8	T2w FLAIR
Vascular network architecture	9	TOF angiography
Iron blood products/calcification	10	Susceptibility weighted images
Water diffusion	11	Diffusion—Fractal Anisotropy (FA)
	12	Diffusion—Trace
	13	Diffusion—Apparent Diffusion Coefficient (ADC)
Haemodynamic	14	Relative local Cerebral Blood Flow (CBF)
	15	Relative local Cerebral Blood Volume (CBV)
	16	Relative local Mean Transit Time (MTT)
	17	Relative local Time To Peak (TTP)
	18	Relative global Cerebral Blood Flow (CBF)
	19	Relative global Cerebral Blood Volume (CBV)
	20	Relative global Mean Transit Time (MTT)
	21	Relative global Time To Peak (TTP)

*(a) Anatomical features were directly acquired by the means of standard static MRI acquisitions that include T1-weighted (T1w) and T2-weighted (T2w) sequences. A Gadolinium-based contrast agent has been used to enhance the signals. (b) Vascular architecture was imaged via beans of Time-Of-Flight (TOF) angiography without the need of contrast agent. (c) Susceptibility weighted images (SWI) have been used to detect the presence of hemorrhages or calcifications. (d) Water diffusion features map the diffusion process of water molecule in tumor and healthy tissue. Diffusion coefficients are the result of the interaction among water and macromolecules or more complex structures, and they are indicators of cell mobility and tumor behavior. (e) Haemodynamic features were acquired by the means of dynamic MRI sequences exploiting the use of Gadolinium-based contrast agent and physiological/mathematical models in both global and local fashion. Features characterize the dynamic of blood supply in terms of volume, flow and velocity and time. Dynamics are indicators of tumor stage, which is related to vascularization's quality (e.g., a very aggressive tumor has a certain vascular system integrated with the healthy surrounded tissue)*.

### 3D Image Domain

A generic 3D image is composed of voxels. A voxel is defined as the smallest measurable cubic volume retrievable from an image and is characterized by three spatial coordinates *(x, y, z)*. These unitary image components allow the value of any measured feature to be computed. In other words, the image space embeds the feature space and a 3D image globally represents the distribution of each measured feature. In our study we have extracted twenty-one 3D features (Table 1) from multimodal MRI acquisitions that were co-registered and re-sliced in order to have uniform voxel dimension (0.37 × 0.37 × 5.5 mm^3^). Each acquisition is targeted to measuring a specific feature. The assembly of all the features forms our dataset. Starting from each voxel *v*, the dataset is obtained from the feature set values *f*_1_…*f*_*N*_ in spatial coordinates:

$$\begin{array}{l} v_{x,y,z}={\left(f_{1},f_{2}\ldots f_{N}\right)}_{x,y,z} \end{array}$$

This means that for a given matrix that is formed in correspondence with each feature extracted from MRI images (a 3D matrix), each voxel includes various feature values, and the feature set (*N* = 21) is obtained from the entire matrix. Therefore, a given voxel is characterized by an array of 21 distinctly informative features.

In-depth image assessment goes through voxel comparison and implies consideration of all values computed from the measured features. Statistically speaking, if two voxels present similar values of features then they have similar behavior in terms of imaging acquisition. From a physiological point of view, this means that the portion of tissue that composes the voxels is also quite similar. Naturally enough, this leads to the idea of applying clustering (say, K-Means or Agglomerative algorithms) to identify groups of voxels with similar values of features, based on the measured correlation (18).

At a methodological level, the higher the number of features and the better the statistics that can be computed, particularly when considering their intra-cluster distribution. Mean, standard deviation, kurtosis, skewness, minimum and maximum can thus be found for each feature.

$$\begin{array}{l} C_{i}\equiv {\left[\begin{array}{c} \ldots .\\ \begin{array}{c} f_{1}^{mean},f_{1}^{std},f_{1}^{kurtosis},f_{1}^{skewness},\;f_{1}^{\min},f_{1}^{\max},\\ \;\ldots .\;\\ f_{N}^{mean},f_{N}^{std},f_{N}^{kurtosis},f_{N}^{skewness},\;f_{N}^{\min},f_{N}^{\max}\\ \; \end{array}\ldots .. \end{array}\right]}_{voxel\;in\;cluster\;i} \end{array}$$

However, we believe that together with the statistics and their informativeness, what counts is contextual to the technology determining the type of features (Table 1), MRI in our case. For this reason, we next introduce networks as our preferred tools for inference. We do not aim to classify images, as in that case DL could be a valid support to radiomic analysis. Our challenge is the interpretability of what we can observe in the image domain once it is translated in a network domain. Notably, topological information becomes available and established properties can be used to quantitatively discriminate among tissue characteristics.

### Network Architecture

A complex network is a graph composed of nodes connected with each other by the means of edges. We built a network in which the edges were determined from the cross-correlation (COR) existing between each couple of nodes, with nodes representing voxel clusters. As common in networks, an adjacency matrix must be built and in our context its generic entry (i, j) is the COR between nodes i and j computed from feature values. These coefficients establish weights assigned to the edges cast between the nodes: a strong correlation implies connection between nodes, which in turn translates into a reduced distance. This relationship depends on the underlying reference network metric. Networks in general present static configurations in which states are associated to conditions and represented by nodes or groups of nodes functionally similar and dynamically inter-communicating (19, 20). Our work analyzes networks derived from images, with configurations of nodes and edges expected to discriminate between cancer and normal image regions.

### Network Topology

**Figure 1:**
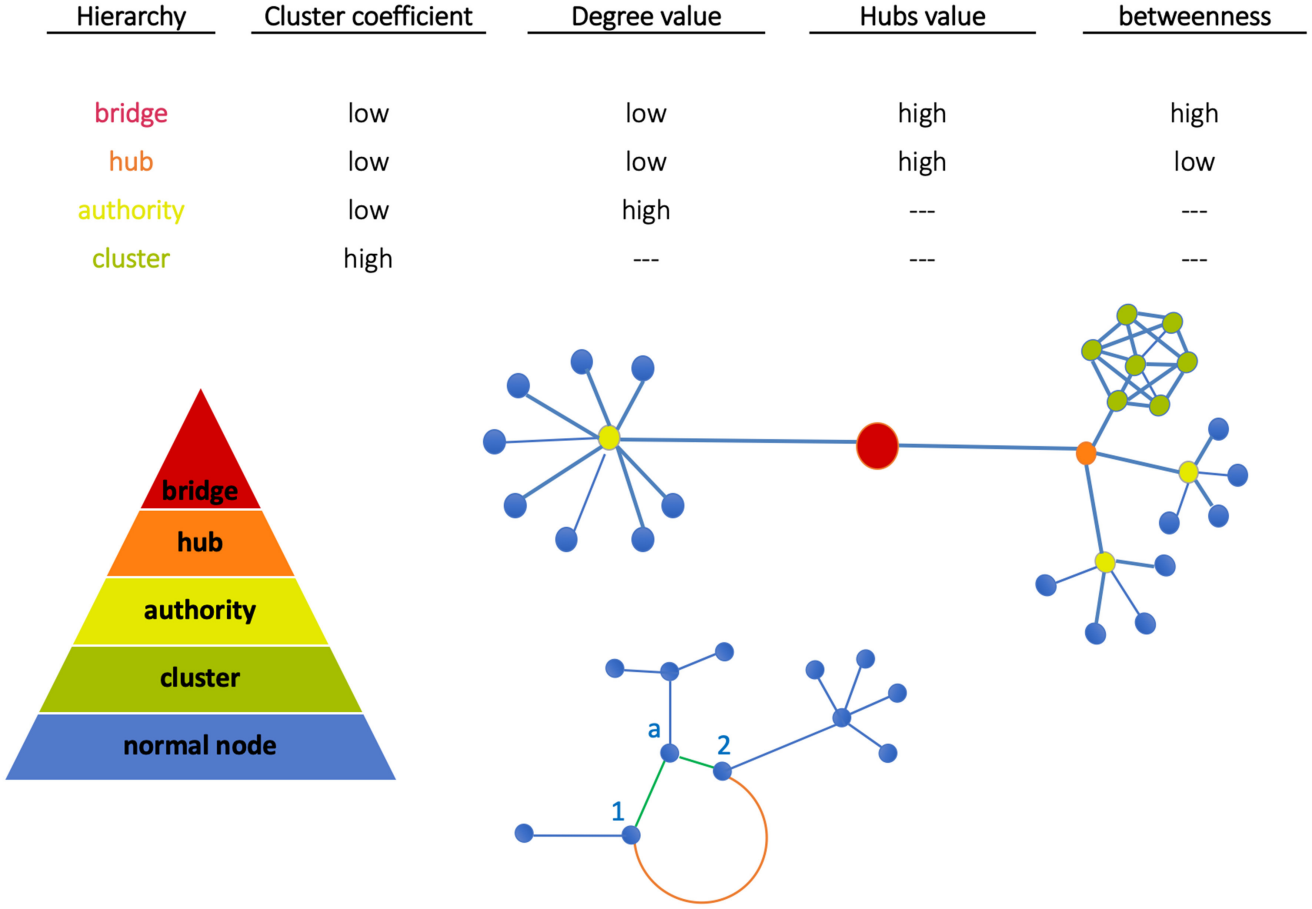
Node classification. Nodes can be grouped all together in a cluster (green) or can be connected individually to an authority (yellow). Similarly, more authorities can be connected together to a hub (orange). The bridge then binds together two subnetworks. At the bottom, Euclidean and geodesic distances are visualized. The nodes 1 and 2 have two kinds of interactions: (a) weak direct interactions (Euclidean distance, orange edge) characterized by a long path, (b) stronger interactions mediated by node a (geodesic distance, green shorter path).

In Figure 1 (top plot) we show a qualitative classification of nodes based on the connections with other nodes. The central plot shows nodes grouped into clusters. Since clusters' density depends on node interconnectivity, each node in the cluster has the same importance. Moving up we find the authority (able to connect multiple nodes) and the hub (binding the authorities). At the top, the bridge links together two or more sub-networks. These are key structures, as in case of depletion of a bridge, the architecture of the network will drastically change since the sub-network will be separated from the rest and the communication between them will be interrupted (21). Finally, sub-networks form communities when significantly connected, i.e., associated by functional similarity.

Node classification between the above structures depends on network metrics. In our work, metrics have been evaluated using the library *NetworkX* (http://networkx.github.io) in Python code and standard network topological properties like clustering coefficient (CC), degree, hubs, betweenness have been measured. Note the following aspects: (a) CC measures the average probability that two neighbors of a node are themselves neighbors (therefore, nodes in image regions uniformly characterized like tumor or normal tissue have the same importance); (b) High-degree nodes are authorities, and hubs assemble multi-connected authorities (image dense spots); (c) High-betweenness nodes, namely the extent to which nodes lie on the path between other nodes, when combined with low-degree properties identify bridges that are relevant for network intra-communication.

In general, each node is to some extent informative about radiomic phenotypes summarized by features, and the key part is establishing associations between nodes that might be possible target. For instance, nodes with feature values characteristic of tissues displaying tumor growth or invasion, and of course integrating these features with genomic ones would allow to explore phenotypically enriched gene signatures (radiogenomics) (22–24).

Apart from the information that nodes deliver, edges too can be classified based on their properties. Through the edge length, the distance between nodes is defined (Euclidean distance):

$$\begin{array}{l} D=1-COR \end{array}$$

**Figure 2:**
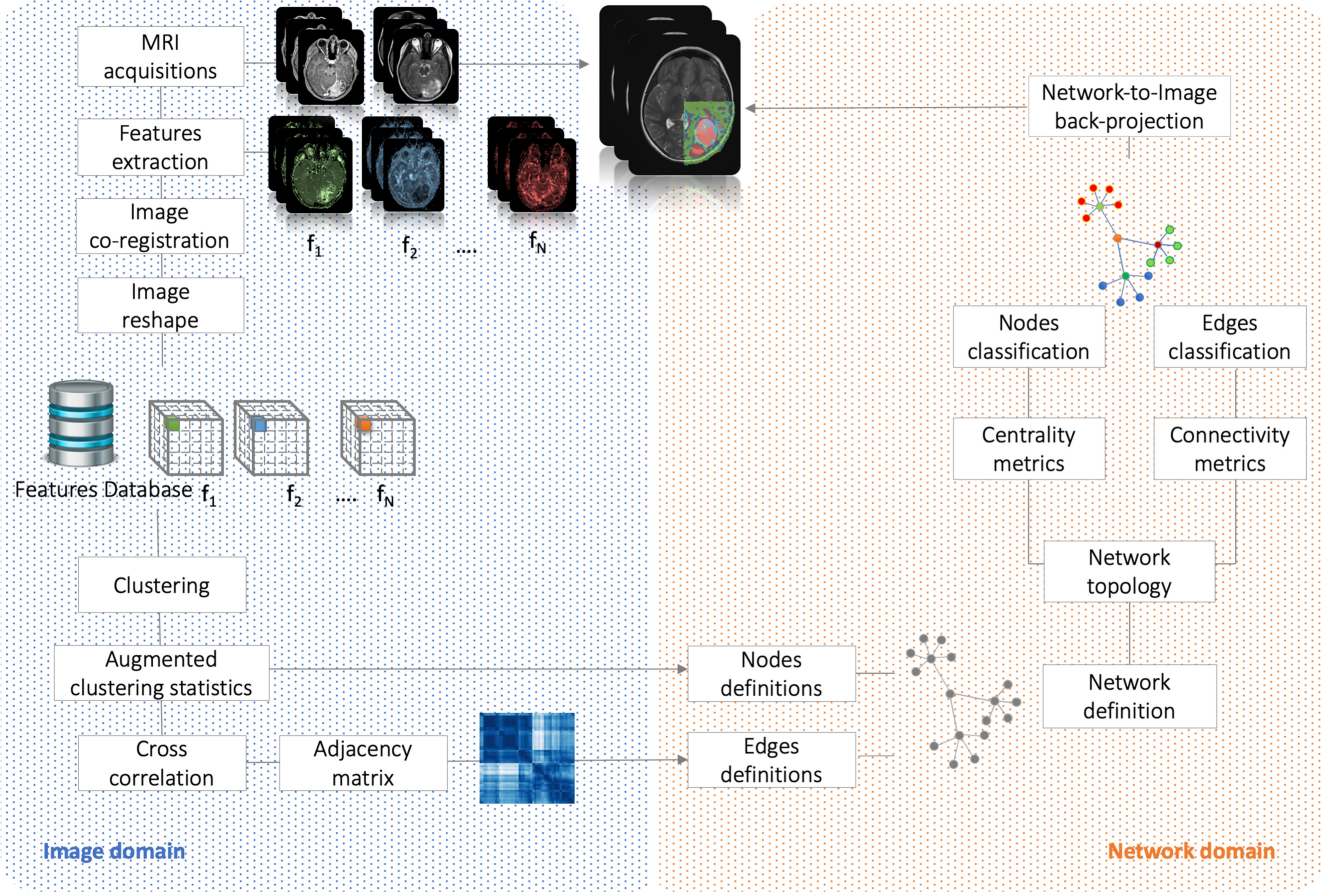
Flowchart. It shows the analysis performed in both image and network domains. Starting from MRI acquisitions, we extracted a dataset of N = 21 features that have been used to group the voxels into clusters and, based on their correlation, to build the complex network. By the means of centrality (nodes classification) and connectivity (edges classification) we have classified nodes and identified subnetworks. Finally, network topology has been back-projected on initial anatomical images.

**Figure 3:**
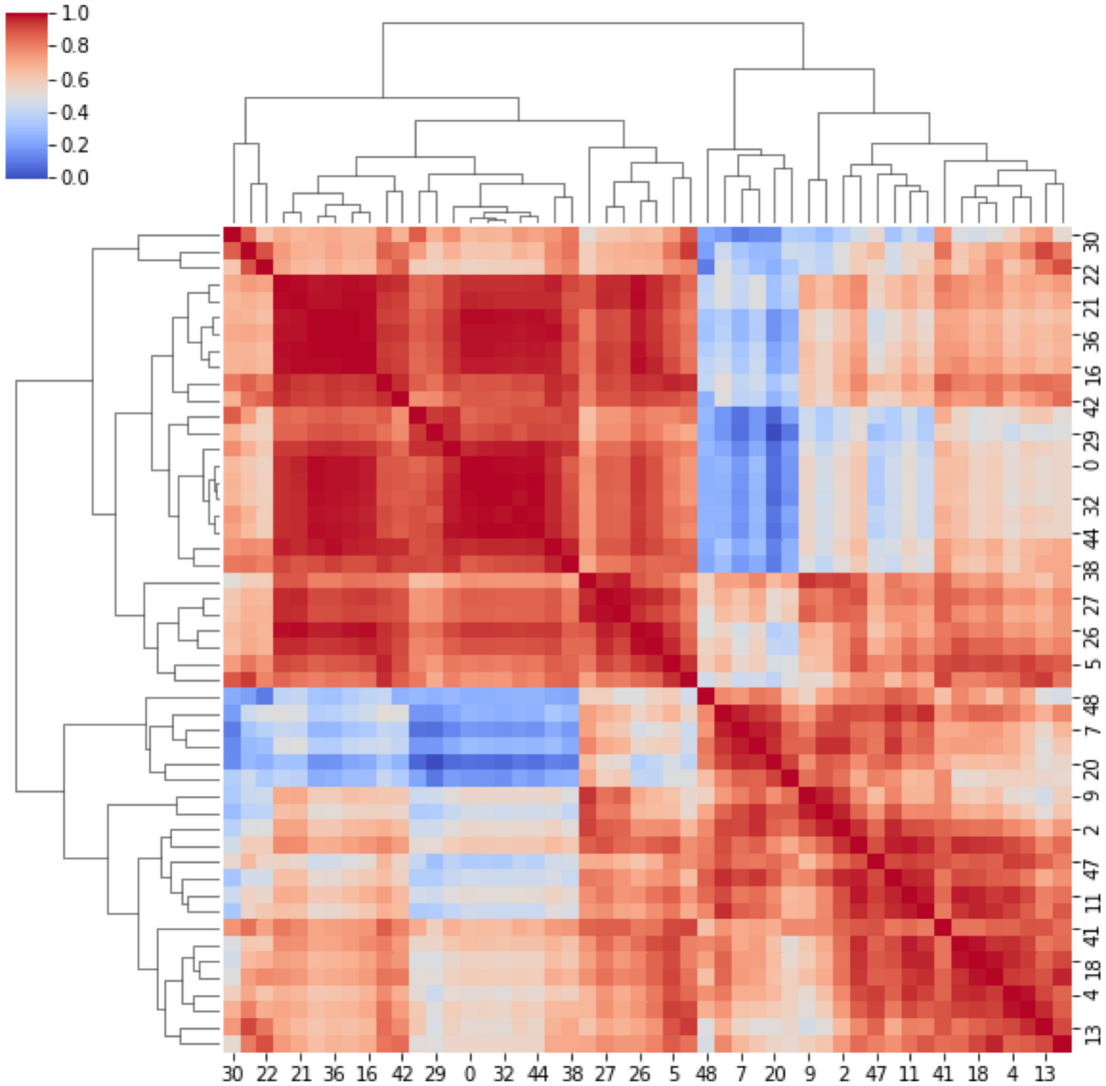
Adjacency matrix. It represents the cross-correlation coefficients between the clusters. The cross-correlation coefficients measure similarity between clusters by varying within a 0–1 range (i.e., from no correlation or no features similarity to strong correlation and identical feature distribution).

Here, strong/weak interaction corresponds to short/long distance, respectively. More importantly, given a destination node, we can infer predictability based on the path length concatenating edges that end at such node. Here, the geodesic distance (shortest path between two nodes) becomes relevant because two nodes can be strong interactors also indirectly (mediated by neighboring nodes). Figure 2 sketches our approach, and Figure 3 displays the computed adjacency matrix. Note that the network-to-image back-projection step implies the possibility to assess the clinical response after therapeutic or surgical interventions or otherwise to monitor patient follow-up.

## Results

### Complex Network

**Figure 4:**
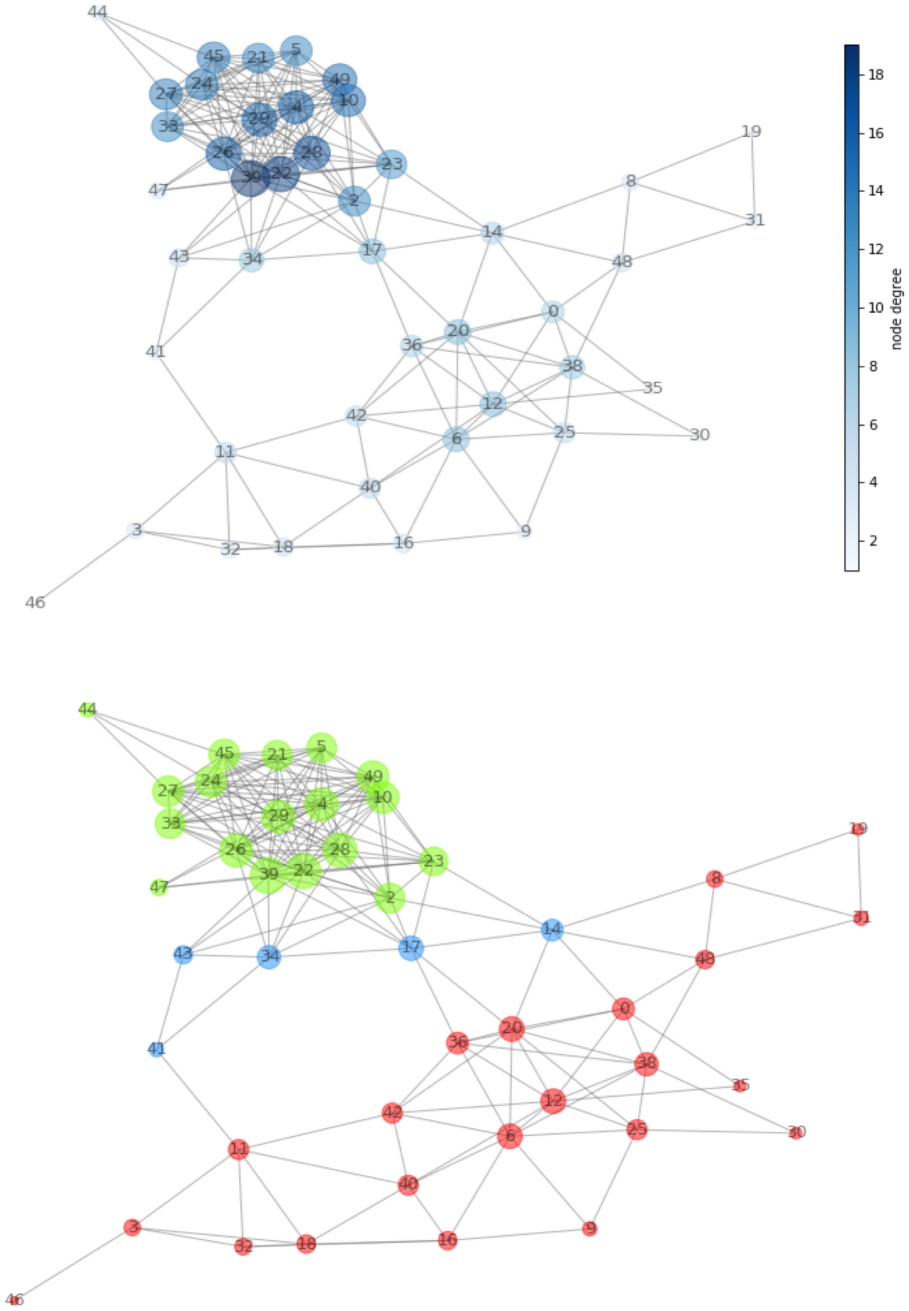
**(Top)** Network created from acquired MRI features (Table 1). The node dimension (and color gradient) is proportional to the degree. **(Bottom)** Network configuration determined by the centrality metric. We have spotted regions with high clustering coefficient and showed them in green and in red. The two are connected together by the means of a set of bridges showed in blue.

A network (Figure 4, top panel) was built for the first patient from the 21 MRI features and consisting of 46 nodes (size proportional to degree) and 209 edges (length proportional to COR). After normalizing the values of the measures defined in Figure 1, we determined the network hierarchy mapped in Figure 4 (bottom panel). For the first patient, a first subnetwork appears densely connected with a cluster structure (green) in which all nodes show high CC value (>0.75).

- A second subnetwork depicted in red without the presence of clusters.
- A group of bridges is depicted in blue and have high betweenness (>0.80) and low degree (<0.20), while connecting the two subnetworks.

Note that the above thresholds are determined based on the available data, following the general principles of limiting network redundancy and maximizing interpretability.

### Tissue Identification

**Figure 5:**
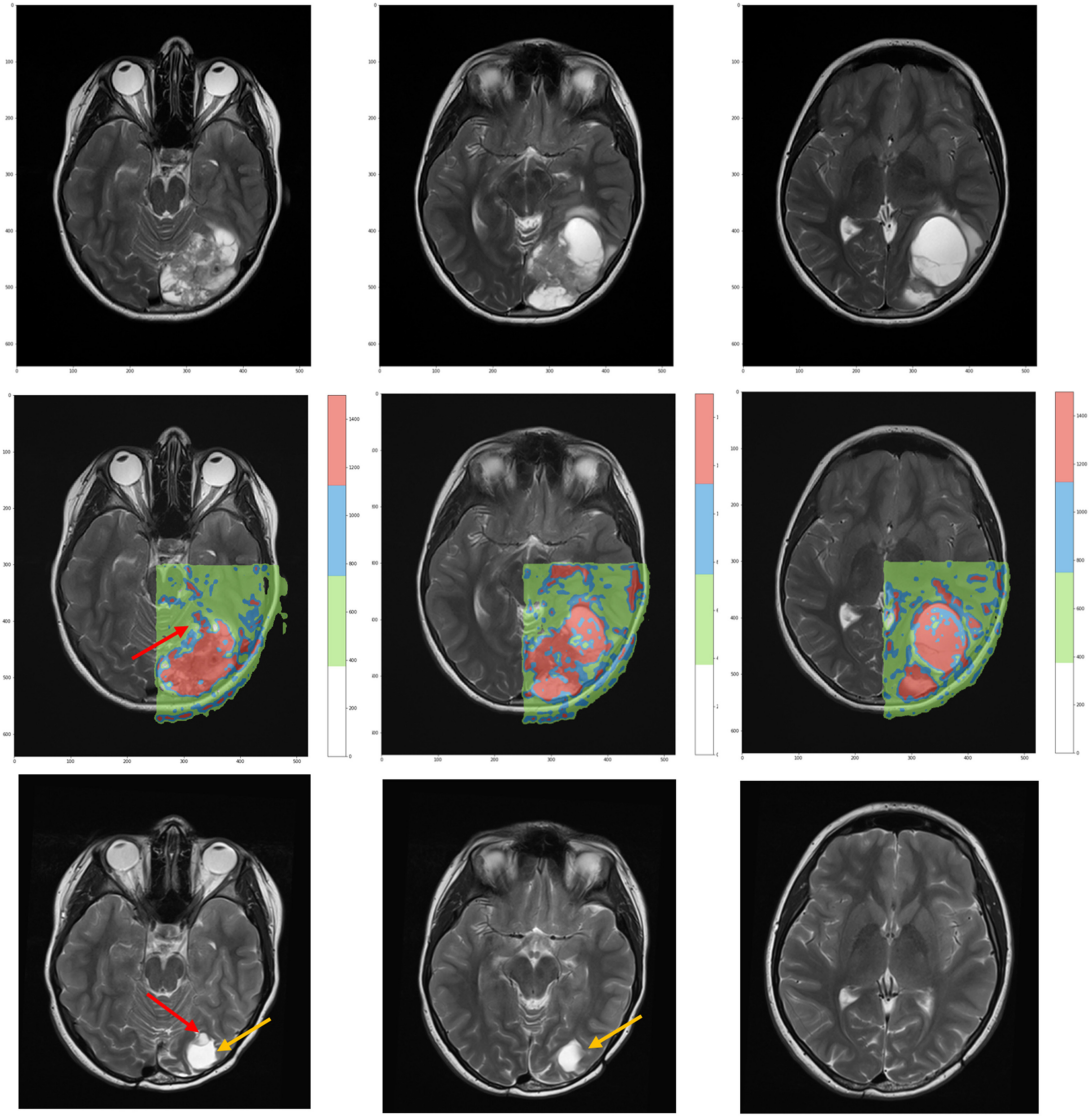
**(Top)** T2-weighted MRI acquisitions showing anatomical details of tumor at time of diagnosis. **(Center)** Overlay of the network classification results, with the red subnetwork covering the tumor and some other external areas. The cluster subnetwork depicted in green identifies the surrounded brain. Finally, the blue bridges linking together the two subnetworks surround the red regions and act as interface between tumor and brain. **(Bottom)** T2-weighted MRI acquired 3 years after surgical and proton therapy treatment highlights the presence of recurrence (red arrow at leftmost plot) and post-surgery cavity (tumor bed, orange arrow at both leftmost and central plots).

According to the color, network nodes of Figure 4 have been back-projected on the T2-weighted image in order to find their anatomical position in the brain. Figure 5 shows that most red nodes fill quite well the bulk tumor while few more spots are localized out of it. The green nodes are located in the healthy part of the brain. Surprisingly, the blue nodes that represent the bridges in the network appear in the images as surrounding the red regions, thus acting at the interface between tumor and healthy tissues.

**Figure 6:**
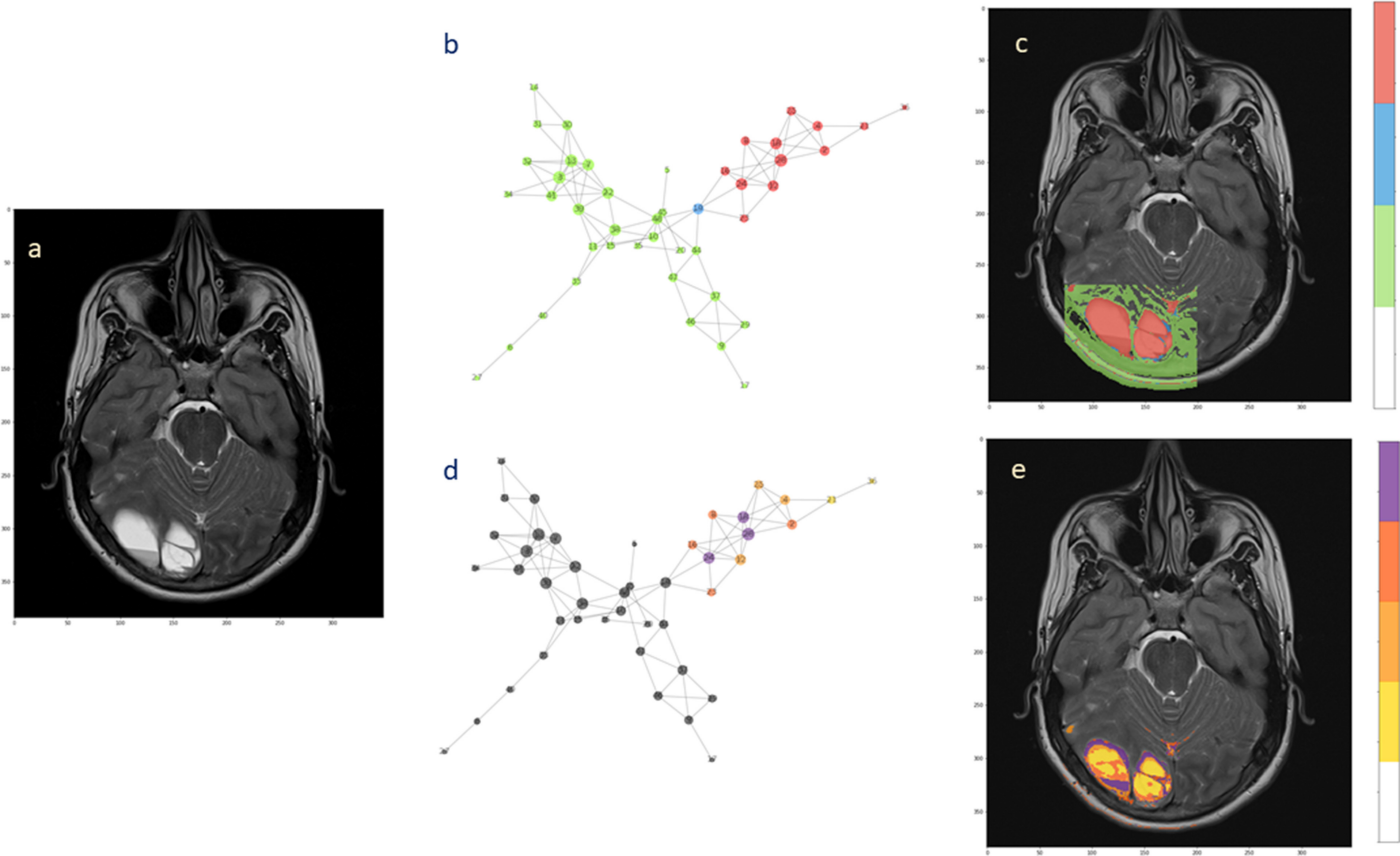
Hierarchical segmentation of intra-tumoral tissue heterogeneity. The extension of the ependymoma is shown in **(a)** the T2-weighted image. **(b)** The complex network obtained from the acquired MRI features by the means of two subnetworks and one bridge node. **(c)** Back-projection onto the anatomical image. **(d)** Hierarchical analysis performed over the tumor subnetworks, with highlighted nodes according to the degree values (from yellow low-degree to violet high-degree). **(e)** Segmentation of intra-tumoral tissues by node hierarchies.

For the second patient, the analysis confirmed the presence of two subnetworks (highlighted in red and green) that copy well with tumor extension and healthy surrounding tissue, respectively (Figures 6b,c). Only a single node (depicted in blue) bridges between the subnetworks. The back-projection onto the anatomical image shows that the corresponding region is smaller and is not overlaying the whole lesion. At present, the small red spot spread in the healthy area can be questioned. These spots could reflect image aberrations, clustering errors or signs of tumor presence. The uncertainty remains based on the physiological features that were measured, indicating presence of tumor. However, there is no clinical evidence that they already represent secondary tumor localizations. They can possibly turn into tumorigenic tissue, although targeted histological examination would be needed, at least in principle.

### Tumor Heterogeneity

Evaluating the node hierarchy in the presence of tumor allowed to stratify the nodes in four groups depending on their degree and then back-project their corresponding voxels onto the T2-weight image (Figures 6d,e). Notably, the tissue identified inside the tumor can be explained by the network topology, showing that the differentiation is possibly reflecting the tissue heterogeneity visible at the anatomical image level (Figure 6a). Especially low-degree nodes cover diffusively the area of the lesion, while high-degree nodes appear at restricted sites. When this happens at the tumor interface, high connectivity could be consequence of well-established phenotypes such as proliferation and invasiveness. Alternatively, this might be considered a mark of increased activity at the tumor microenvironment level.

### Network Identification

**Figure 7:**
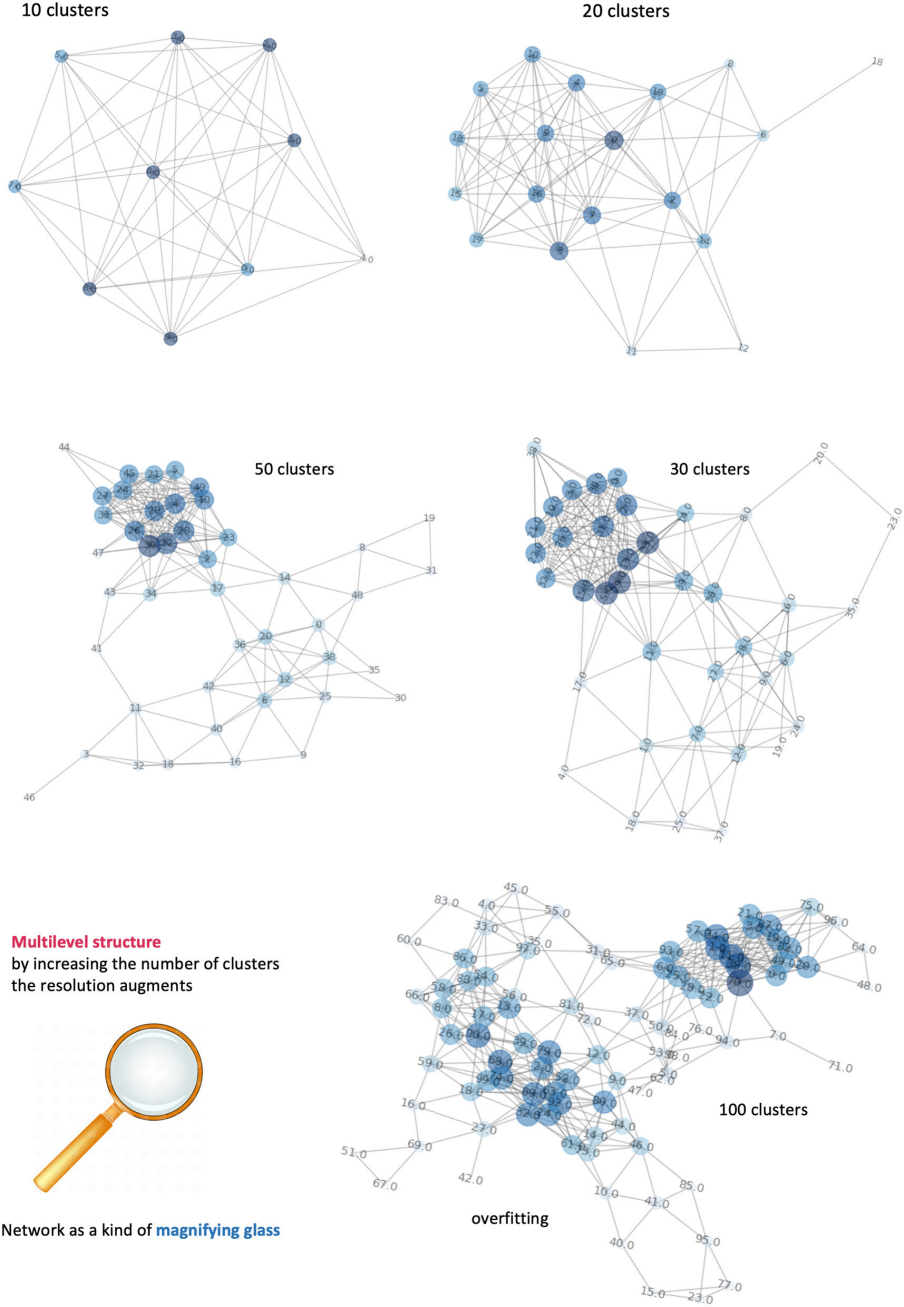
Dependence of networks' structure on clusters' number. It is possible to visualize different levels of the network by incrementing the number of clusters. Equivalently, an increase of network's complexity allows better definition of the number of tissues and their connections. When the number of nodes exceeds the informative content provide by our features, the network is overfitting and the links between nodes follow a random process.

As in most of clustering procedures, the exact number of clusters is not known *a priori* and must be estimated. However, we consider this clustering only a pre-processing step in voxel domain, and the control over the clustering quality can be done by iteratively (a) incrementing the number of clusters and (b) visualizing the respective network configurations. Figure 7 displays a sequence of increasing clusters/nodes number clearly showing that already 20 clusters/nodes make the network structured, and then further detailed with 30 and 50 clusters. In principle, increasing the resolution limit means considering smaller and smaller voxel groups, at clear risk of overfitting (limited representativeness of physiological features). Because of such information gap, 100 clusters indicate edges that are artificially present as no longer robust and mainly dominated by the law typical of a random process. We have estimated such limit to be reached at around 60 clusters, and thus used 50 clusters as stopping criterion. In conclusion, the clustering classification's aim is to identify voxels with similar features, or that belong to the same kind of tissue. Clusters are then formed from similar voxels and represented as network node**s**. Importantly, this mapping preserves the original spatial resolution.

## Discussion

### Role of Networks

Biomedical data are rich in structure (modular) and systematically organized. A natural question is whether hierarchies are identifiable according to specific relationships between data attributes. Networks include such hierarchies, and the higher elements (hubs and central nodes) are the ones influencing the overall behavior (25, 26). When considering tumor's behavior, two categories of nodes have high importance: (a) *Bridges or sub-network connectors*; (b) *Authorities or large-scale node aggregators*. In the latter case, even if located in the middle of the pyramid, authorities regulate a large tissue part, and thus act as global influencers or regulators. In principle, from a clinical point of view, bridges and authorities could be considered the main targets for therapy due to their role in connecting and controlling tumor tissues.

We reported specifically on the application of complex networks for tumor characterization with two main outcomes: (a) Identification of the interface between tumor and healthy surrounded tissue, and (b) Segmentation of intra-tumoral tissue heterogeneity based on node hierarchies. By focusing on response to therapy (relapse or not of ependymoma) we showed clearly identifiable back-projection effects from the observed network hierarchy. The anatomical images have clearly identified (red region) the extension of the tumor location separated from the healthy brain (green region). However, as smaller regions (blue ring) surrounded the identified tumor regions (red spots), a match was established between these spots and network bridges (27).

In general, no clear conclusions hold for the small regions located in the apparently healthy brain. Based on images readouts such regions show similar features and behave similarly to tumor. Whether this is sufficient to hypothesize a likely relapse deserves further investigation and probably richer sequence of data. The availability of more patients would facilitate the goal of designing targeted risk tools. However, PM data present nowadays so many dimensions at the individual patient scale that their integration has become a need. Such aggregate would involve all the features spanning multiple domains (environmental, genetic, clinical, therapeutic, follow-up and lifestyle, nutrition etc.) and informing on disease phenotypes. For instance, observation of intra-heterogeneity occurring along the disease course might reveal molecular target expression levels objectively limited in the ability to guide treatment (what are suitable cutoffs for assessing the presence of targets? How is characterized the target expression at time of recurrence?).

These knowledge gaps call for more precise prevention, detection and treatment, and in turn requires a clinically-focused effort by computational oncologists. Some key challenges have been identified in multiple data modalities, insufficient data, model interpretability and learning methods (28). In this work we have addressed mainly the second and third of these challenges, given the availability of images and the centrality assigned to network inference.

### Role of Cancer

We have selected two cases *ad hoc* and showed the utility of retrieving features that can be put in relationships by a simple network metric. Then we have assessed the validity of such features in view of the patient history, i.e., whether or not a recurrence occurred years after the surgical and proton treatment (Figure 5).

Concerning model interpretability, a limiting factor is how to measure the effects of therapy at the image level. The anatomical changes due to the surgical resection prevent from an exact localization of the recurrence in the diagnostic images. Nevertheless, we identified such region as the part of brain in contact with the superior right part of the primary tumor that moved down after tumor removal (Figure 5, central panel). Based on the anatomical images, such regions seem located in the healthy tissue. By contrast, the network assigns it to the red spots associated with the primary tumor. This means that although at the time of diagnosis the risk of recurrence is hard to predict, networks can be highly informative for treatment planning and follow-up.

Our results are also relevant to tumor microenvironment. First, an interface between tumor and healthy tissue is clearly identified by network bridges. Second, highly complex interactions between tumor and host tissue occur exactly here, something relevant for clinical decisions and intervention. From a tumor perspective there is the need to degrade the extracellular matrix and change pH to promote cellular infiltration, while from the host there is the urgency to promote immune response to block expansion and inoculate lymphocytes into the tumor (29, 30). The success of chemo- and radiation therapy strongly relies on the extension and vascularization of such interface. As a matter of fact, any systemically administered drug needs to cross such interface to reach the tumor. Furthermore, a good oxygen perfusion, which is necessary for the radiation therapy to be effective, is determined by the vascularization of such interface (31). On the other hand, the same factors helping tumor treatment may also increase the probability for tumor cells to detach and enter the blood flow, which translates into an increase of metastatic potential.

### Methodological Choices

We applied a (non-deep) network approach. MR imaging features have been used to establish attributes and dimension of network nodes (or voxel clusters) with reference to ependymoma patients monitored for response to proton therapy and subject or not to relapse. For the challenge of image classification, the standard practice is to adopt DL methods to dissect images and define class characteristics. DL has been widely used in brain studies, especially with MRI. The idea is to learn sequentially, from local patches to representations and then labels. However, a limitation is a certain lack of contextual information required for some task. This needs further testing and makes DL suboptimal for a content-based image retrieval task. Also, in some cancers large datasets are missing and class imbalance may bias the results. In particular, the size variation of target object within the image is very relevant to our scopes. DL training with multiscale input data followed by output data fusion is currently non-sufficiently informative about robustness to size variation.

In our network approach to images, node connectivity could be of high importance in clinical perspective. Let us consider spatio-temporal aspects. For example, let us consider two tissues apparently disconnected, namely an inner region of the tumor and a portion of healthy tissue far from it. Even if they are not in contact, or spatially contiguous, these two regions can still strongly interact through vessels. In principle, estimating the distances between nodes along the entire network allow to discriminate between short- vs. long distance processes, which are represented by tumor infiltration and metastases formation, respectively. Temporality is a key factor, as for instance relapse has been documented in one of the examined cases. Explicit consideration of this factor supported by quantitative assessment and visual interpretability of results adds value to the arguments of those emphasizing the need to investigate longitudinal heterogeneity, possibly therapy-driven along disease course. Accounting for follow-up information means covering with the analysis multiple timepoints and capture dynamic aspects of cancer phenotypes.

## Concluding Remarks

### Challenges

The integration of medical imaging data is traditionally a complex task. Each set of data is a measure of the activity of multiple biological and physiological processes. This means that a synthesis between two images is not necessarily informative by itself, i.e., by simply joining component images. Without integrating the unitary components, the synthesis would be incomplete and the image fusion not accomplished. Aware of such limitation, we proposed a biomedical imaging model centered necessarily on voxels as the unitary components. The strategy of integration is inspired by network inference through a few landmark points:

- *Cancer data are extremely heterogeneous*. This is visible from both the data generating sources, which in turn calls for different (complementary or not) techniques to treat them (32, 33). Networks can work with a blend of underlying machine learning algorithms, data fusion methods and statistical principles.
- *Voxels acquire meaning when connected with each other*. It is expected that tissue characteristics are reflected by the voxels and they clusters, but the picture is far more blurred with cancer microenvironment. Therefore, voxels and their clusters inform of tissue characteristics more or less distinctly depending on how similar they are to each other.
- *Images can be represented by sets of highly specific features*. For instance, physiological processes cover different spatio-temporal scales, and require many parameters to be accounted for by models. Metabolism-driven images refer metabolic processes taking place at the molecular level. Instead, blood perfusion takes place at the cellular level. Biological processes are interrelated and organized sequentially or hierarchically (34). In all such cases features have different properties that need to be harmonized, and networks offer scaffolds suitable to do so.
- *Stochasticity and Dynamics must be accounted for*. This translates into the need of considering the effects of small-to-big interactive perturbations that may induce drastic changes and propagate their influence in complex non-linear systems (35, 36).

### Precision in Radiomics

While DL has been applied to a myriad of biomedical imaging contexts, including onco-radiomics, complex networks have been employed to a much lesser extent in medical imaging applications, despite they can cope quite well with most of the previously described hurdles. This gap is due to several factors. For classification, reconstruction, recognition and similar tasks DL is now a gold standard. The problems with DL, together with overfitting, are mainly interpretability and transparency, both dependent on the “black box” learning paradigm.

At the other end, networks are composed of nodes bound together by edges. Such simple geometrical representation offers properties that fit most needs, such as (i) Leveraging a hierarchical structure (ii) Allowing a multi-layer architecture describing different spatial scales (iii) Offering a template use for modeling external perturbations aimed at simulation of treatments and interventions and (iv) Providing built-in integration between image data and other molecular, genetic, clinical data. Network topological properties highly stimulate our aim of demonstrating both principled and practical applicability of network inference to medical images together with their radiomic integrability.

Radiomics elaborates the informative contents of big data associated to medical images and obtained in relation with physiological, clinical, biological, genomic evidences. This merge necessarily generates a series of novel tasks in the clinical practice and in the scientific research designed to support clinical decisions. Quantitative imaging has many tools and methods available to bring added value from the translational medicine standpoint. The expected gain is that data (including images) volume and variety may lead to superior accuracy in measurements and more objective data interpretation.

Precision radiotherapy is the associated field that may receive immediate benefits from the availability of imaging-integrated diagnostic tools useful for therapy selection and response assessment (37). Method standardization is a requirement for applications across multiple centers and in prospective clinical trials so to establish the essential role of novel imaging biomarkers. Networks present good properties in such regards, as they are easily scalable and generalizable to various data types integrable with medical images. Theranostics is another promising area of research in which network-guided inference may find application, especially for an understanding of the efficacy of newly delivered drug classes (38).

### Limitations of the Approach

A few bottlenecks emerged from our work. First, the need of understanding better physiological vs. pathological implications of network nodes. This calls for further research into data knowledge assimilation and representation involving several features, from images and other data types that might be assessed as integrated node attributes. Second, the need of newly conceptualized designs of therapy simulations, which might find in our approach a natural application ground and an efficient computational framework. A third direction that needs further work involves theoretical developments in complex networks potentially inspiring knowledge integration and modeling from data outsourced by medical multimodal imaging and radiomics. In all such directions, our work is continuing with applications over larger patient cohorts and generalization across cancers.

## Data Availability Statement

All datasets generated/analyzed for this study are included in the manuscript.

## Ethical Statement

This case-study was reviewed and approved by Ethikkommission Nordwest und Zentralschweiz on February 28, 2018, License number: EKNZ 2019-00346.

- Written informed consent was obtained from the participant's parents for participation in the scientific study.
- Written informed consent was obtained from the participant's parents for the publication of this case and any potentially identifiable data. Written informed consent to participate in this study was provided by the participants' legal guardian/next of kin.

## Author Contributions

MD and EC conceived the methodological approach and wrote the paper. MD performed imaging and computational analyses. AP and MD characterized the ependymoma case. AP, SS, AL, and DW supervised the radiological work. All authors read and approved the manuscript.

### Conflict of Interest

The authors declare that the research was conducted in the absence of any commercial or financial relationships that could be construed as a potential conflict of interest.
